# Variations in the impact of the new case-based payment reform on medical costs, length of stay, and quality across different hospitals in China: an interrupted time series analysis

**DOI:** 10.1186/s12913-023-09553-x

**Published:** 2023-06-02

**Authors:** Xue Tang, Xinyu Zhang, Yajing Chen, Jiaqi Yan, Mengcen Qian, Xiaohua Ying

**Affiliations:** 1 School of Public Health, Fudan University, Shanghai, China; 2 Key Laboratory of Health Technology Assessment (Fudan University), Ministry of Health, Shanghai, China

**Keywords:** DIP, Total expenditure, Length of stay, In-hospital mortality, Different hospitals

## Abstract

**Background:**

In 2018, an innovative case-based payment scheme called Diagnosis-Intervention Packet (DIP) was piloted in a large developed city in southern China. This study aimed to investigate the impact of the new payment method on total medical expenditure per case, length of stay (LOS), and in-hospital mortality rate across different hospitals.

**Methods:**

We used the de-identified patient-level discharge data of hospitalized patients from 2016 to 2019 in our study city. The interrupted time series model was used to examine the impact of the DIP payment reform on inflation-adjusted total expenditure per case, LOS, and in-hospital mortality rate across different hospitals, which were stratified into different hospital ownerships (public and private) and hospital levels (tertiary, secondary, and primary).

**Results:**

We included 2.08 million and 2.98 million discharge cases of insured patients before and after the DIP payment reform, respectively. The DIP payment reform resulted in a significant increase of the monthly trend of adjusted total expenditure per case in public (1.1%, *P* = 0.000), tertiary (0.6%, *P* = 0.000), secondary (0.4%, *P* = 0.047) and primary hospitals (0.9%, *P* = 0.039). The monthly trend of LOS increased significantly in public (0.022 days, *P* = 0.041) and primary (0.235 days, *P* = 0.032) hospitals. The monthly trend of in-hospital mortality rate decreased significantly in private (0.083 percentage points, *P* = 0.002) and secondary (0.037 percentage points, *P* = 0.002) hospitals.

**Conclusions:**

We conclude that implementing the DIP payment reform yields inconsistent consequences across different hospitals. DIP reform encouraged public hospitals and high-level hospitals to treat patients with higher illness severities and requiring high treatment intensity, resulting in a significant increase in total expenditure per case. The inconsistencies between public and private hospitals may be attributed to their different baseline levels prior to the reform and their different responses to the incentives created by the reform.

**Supplementary Information:**

The online version contains supplementary material available at 10.1186/s12913-023-09553-x.

## Background

The continuous increase in health expenditure is a challenge faced by all healthcare systems [1]. Fee-for-service (FFS) is a typical retrospective payment system under which hospitals are remunerated according to the number of services provided. Over time, various countries found that doctors tend to apply more procedures to increase their income under FFS, which leads to wasted medical resources and unjustified increases in health costs [2]. To control the increasing hospital expenditure, some medical insurance payment systems switched from retrospective payment to the prospective payment system (PPS) [3]. Case-based payment is a typical type of PPS, which integrates patients with similar diagnoses, similar clinical processes, and resource consumption into limited case groups, under which hospitals are remunerated according to the case group [4].

In response to rising healthcare costs and to regulate provider behaviors, China developed a new case-based patient classification payment system in the context of the rapid development of informatization and the accumulation of big data in hospitals, which is called Diagnosis-Intervention Packet (DIP). It is important to note that DIP is a novel case-based payment system that differs from the traditional Diagnosis Related Groups (DRGs) payment system. Firstly, the patient classification rules of DRGs require high-quality medical record home page data and judgment from physicians, so the roll-out of DRGs has some challenges in middle-and-low-income countries with limited resources. The DIP classification circumvents the major challenges of DRGs due to lower data quality requirements and less reliance on physicians’ judgment. Compared to the DRGs payment system, the DIP payment system primarily classifies inpatients by combinations of the first four digits of principal diagnosis codes (International Classification of Diseases, 10th Revision) and procedure codes (International Classification of Diseases, 9th Revision, Clinical Modification), without considering patient demographic and administrative characteristics (such as age, gender, complications and comorbidities, discharge status, etc.). Secondly, DRGs grouping results are typically less than 1000 case-based groups, whereas DIP grouping results are more than 10,000 case-based groups. Subsequently, relative weights (RW) are assigned to each DIP group based on the historical average relative resource utilization of diagnosis and treatment. The DIP payment system determines basic RW according to the relative costs for the same DIP group between different levels of hospitals. The RW of the same DIP group is higher in high-level hospitals with more resource consumption. Additionally, extra RW is added to hospitals with a higher Case Mix Index and key disciplines, which is an improvement to encourage high-level hospitals to treat severely ill patients [5, 6]. Thirdly, unlike the traditional DRGs payment system in which the absolute reimbursement for each group is predetermined, the monetary value for each RW point of the DIP payment system is floating, depending on the annual regional global budget and the sum of RW points for all inpatient cases. Further details were introduced in a previous study [6].

We selected a large and developed city in southern China as our study sample, one of the first cities to pilot the DIP payment reform in all hospitals for inpatient services since January 2018. Before the pilot DIP payment reform, the city implemented a strict cost control policy that the medical security bureau assigned a fixed and same rate for each admission of insured inpatients in medical institutions, and set a compensation limit for each hospital in its jurisdiction. As a result, hospitals tend to reject patients when hospitals reach the ceiling of annual compensation. In fact, the medical security bureau usually compensates hospitals appropriately for exceeding the limit. In addition, the growth rates are set for the “fixed-rate per admission” and “compensation limit for each hospital” based on the historical cost data. After the reform, “fixed-rate per admission” was converted to DIP payment system, and the “compensation limit for each hospital” was replaced by the city-level regional global budget, so that hospitals themselves need to compete fairly for the regional global budget regardless of the level or ownership of hospitals. Therefore, hospitals may be inclined to adopt advanced technologies and treat severe patients to compete for DIP groups with high RW after the DIP reform.

Existing literature has indicated that the implementation of DRGs or similar case-based payment systems may have varying impacts on hospitals with different characteristics, such as level, ownership, type, teaching status, and others [7, 8]. Studies from Taiwan have shown that patients in private hospitals had a shorter length of stay (LOS) than those in public hospitals under the case-based payment system [7, 8]; however, a study conducted in Poland indicated the contrary [9]. Studies conducted in China proved that DRG-based payment has different impacts on total expenditures and LOS across hospitals with different levels [10–12]. Additionally, studies conducted in Korea showed that implementing the DRG-based payment led to significant reductions in LOS and mixed findings on costs in general hospitals [13, 14]. Nonetheless, most of these studies used a multiple regression analysis to analyze the relationship between LOS, medical costs, and hospital characteristics under DRGs or similar case-based payment systems. Few studies have employed the interrupted time series (ITS) method to examine the changes in indicators across different hospitals before and after the DIP reform.

We hypothesized that the DIP payment reform has created different incentives for healthcare providers, resulting in varied responses across different hospitals. Firstly, hospitals may improve efficiency to save costs after the DIP payment reform, to gain more benefits, especially private hospitals aiming at maximizing benefits. Secondly, compared with private hospitals, public hospitals might experience an increase in total expenditure per case as they focus on developing key disciplines by treating severe patients requiring advanced technologies and high treatment intensity. Thirdly, compared with low-level hospitals, high-level hospitals might experience even higher increases in total expenditure per case after the reform, as they treat more severe patients requiring high treatment intensity and advanced technology and medical resources.

As an innovative medical payment system in China, the actual impact of DIP payment reform across different hospitals lacks empirical evidence. Therefore, this study aims to provide evidence on the impact of implementing DIP payment reform under the regional global budget on adjusted total expenditure per case, LOS, and in-hospital mortality rate in hospitals with different characteristics in one of China’s earliest and largest piloting cities. Our findings will provide policymakers with empirical evidence from different hospitals to expand the DIP payment reform.

## Methods

### Data and sample

We used the de-identified patient-level discharge data of hospitalized patients in all hospitals in the city from 2016 to 2019. The dataset contains individual-level information of patient characteristics, diagnoses, procedures, expenditures, etc. Further, it includes hospital-level characteristics of ownership (public or private) and accreditation level (tertiary, secondary, and primary, determined by the size and capacity of hospitals [15]). These two hospital features are independent from each other, which means that both public and private hospitals could be tertiary, secondary, or primary. We included hospitalized adult patients covered by the social basic health insurance scheme, and the scheme covered 12.48 million people (84% of the population in the city) in 2018 [16].

### Study variables

We included total expenditure per case, LOS, and in-hospital mortality rate as the main outcome variables to measure the efficiency and quality of healthcare. Total expenditure was adjusted to 2019 considering inflation using the annual consumer price index of China [17]. LOS was calculated by the difference between the admission and discharge dates of each hospitalization. In-hospital mortality was identified by the variable “discharge status” in the dataset and was constructed as a dummy variable at the discharge level. To further understand the incentive and impacts of the DIP reform, we also observed the number of discharge cases and relative weight per case before and after the reform. Relative weights of hospitalized patients were produced by assigning each case a DIP group and its corresponding RW using the DIP classification system in 2018, which makes the RW comparable across different years. Patient age (continuous variable at the patient level), sex (male or female), and comorbidity severity reflected by the Charlson Comorbidity Index (CCI) [18, 19] were included as control variables.

### Statistical analysis

Patient characteristics and outcome variables were compared before and after the DIP reform in the overall sample, by hospital ownership and by hospital level using t-test and chi-square test. We then performed an interrupted time series (ITS) analysis with a segmented regression model to compare monthly trends of three outcome variables in different hospitals before (January 1, 2016, to December 31, 2017) and after (January 1, 2018, to December 31, 2019) the DIP payment reform. The model was specified as follows:

1 $${\textrm{Y}}_{\textrm{t}}={{\upbeta }}_{0}+{{\upbeta }}_{1}{\textrm{T}}_{\textrm{t}}+{{\upbeta }}_{2}{\textrm{DIP}}_{\textrm{t}}+{{\upbeta }}_{3}{\textrm{DIP}}_{\textrm{t}}{\textrm{T}}_{\textrm{t}}+{\upalpha }{\textrm{X}}_{\textrm{t}}+{{\upepsilon }}_{\textrm{t}}$$

where ${\textrm{Y}}_{\textrm{t}}$ represents the aggregated outcome variable in month $\textrm{t}$. Total expenditure per case was logarithmically transformed in the model considering its skewed distribution. T_t_ is a monthly time during the study period. We exclude data of three months from October to December in 2017 before the reform in the analysis, considering the relatively more missing data and small sample size in the original dataset, and thus the abnormal value of aggregated outcomes in these three months. ${\textrm{DIP}}_{\textrm{t}}$ is a dummy variable, which equals 0 before the DIP reform and 1 after the reform. ${\textrm{X}}_{\textrm{t}}$ is a vector of the control variable, including the number of discharge cases, average age, proportion of male sex, average CCI, and seasonality. The intercept ${{\upbeta }}_{0}$ represents the baseline level of the outcome variable, and ${{\upbeta }}_{1}$ is the monthly slope (trend) of the outcome variable before the DIP reform. ${{\upbeta }}_{2}$ and ${{\upbeta }}_{3}$ are changes in the level and slope of the outcome variable after the reform, respectively.

We fitted a Prais-Winsten estimation with the Durbin-Watson statistic to adjust for autocorrelation and used robust standard error [20, 21]. We first conducted ITS analysis for hospitalized patients in all hospitals. Subsequently, we separately analyzed the impacts of the DIP reform in different ownerships (public and private) and levels (tertiary, secondary, and primary). In the robustness checks, we also added hospital level as a covariate in the analyses of different ownerships and added hospital ownership as a covariate in the analyses of different levels. We set 5% as the threshold of statistical significance. Stata 16.0 for Windows was used in all analyses.

## Results

### Sample characteristics

Our analysis included 2.08 million and 2.98 million discharge cases of insured patients before and after the DIP payment reform, respectively. Table 1 shows the characteristics of our study sample. The average age of patients was roughly 59 years, with male patients accounting for approximately 45%. We included 235 public hospitals and 59 private hospitals. By level, we included 90 tertiary hospitals, 92 secondary hospitals, and 126 primary hospitals. Discharge cases in public and tertiary hospitals accounted for approximately 95%, and 80% of the whole sample, respectively. The unadjusted level of total expenditure per case increased significantly (*P* = 0.000) after the DIP reform, while the average LOS and in-hospital mortality rate both decreased significantly (*P* = 0.000). Sample characteristics by hospital ownership and hospital level are presented in Table S1 and Table S2 of the supplementary appendix. Specifically, most patients were from tertiary hospitals in both public and private hospitals, although the proportion of patients in secondary and primary hospitals was larger in private hospitals. The overwhelming majority of patients were from public hospitals in each hospital level, while the proportion decreased from tertiary to primary hospitals.

**Table 1:** Sample characteristics of hospitalized insured patients, 2016–2019

Variables	Before DIP reform, 2016–2017	After DIP reform, 2018–2019	*P* value
**Patient characteristics**			
Discharge cases, No.	2,077,155	2,983,971	
Age, mean (SD)	58.41 (18.23)	58.67 (18.20)	0.000
Male sex, No. (%)	934,407 (44.98)	1,358,316 (45.52)	0.000
Charlson Comorbidity Index, mean (SD)	0.76 (1.34)	0.93 (1.50)	0.000
Hospital ownership, No. (%)			0.000
Public (*N* = 235)	1,988,492 (95.73)	2,832,538 (94.93)	
Private (*N* = 59)	88,663 (4.27)	151,433 (5.07)	
Hospital level, No. (%)			0.000
Tertiary (*N* = 90)	1,670,723 (80.43)	2,365,135 (79.26)	
Secondary (*N* = 92)	317,308 (15.28)	431,623 (14.46)	
Primary (*N* = 126)	89,124 (4.29)	187,213 (6.27)	
**Patient outcomes**			
Total expenditure per case, mean (SD), RMB	15445.33 (19540.18)	15915.35 (19409.33)	0.000
Length of stay, mean (SD), d	9.59 (11.37)	9.22 (11.74)	0.000
In-hospital mortality, mean (SD), %	1.20 (10.90)	1.09 (10.39)	0.000

DIP denoted the Diagnosis-Intervention Packet payment reform; N the number of hospitals. Total expenditure was adjusted to 2019 considering inflation using the annual consumer price index of China

### Total expenditure per case

**Fig. 1:**
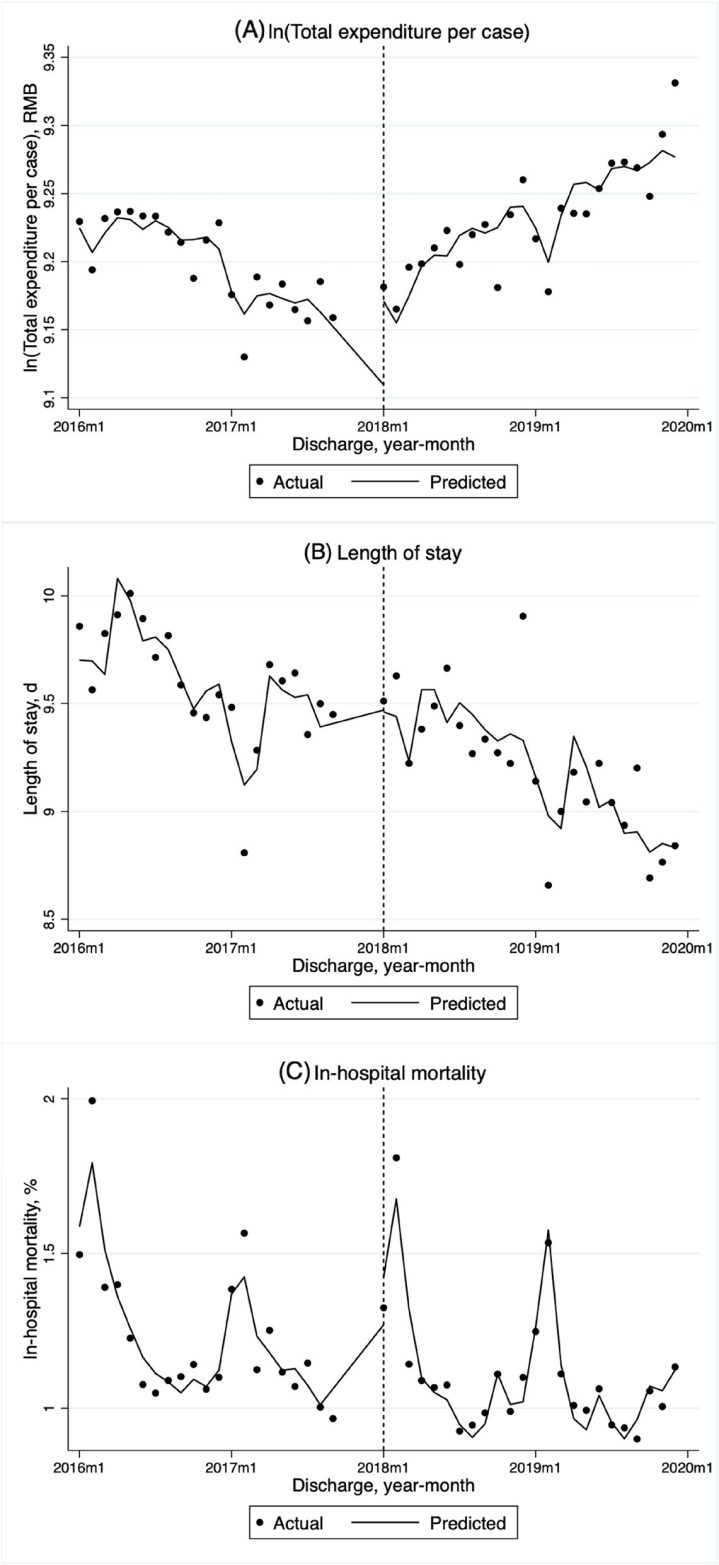
Monthly trends in adjusted total expenditure per case (in log form), length of stay, and in-hospital mortality rate of hospitalized insured patients. Notes: **A** Monthly trends in adjusted total expenditure per case (in log form and were adjusted to 2019 considering inflation using the annual consumer price index of China). **B** Monthly trends in the adjusted average length of stay. **C** Monthly trends in adjusted in-hospital mortality rate. The vertical dashed line denotes the implementation of the DIP payment reform on January 1st, 2018. The solid trend line is predicted based on segmented regression of the time series model (before the reform: January 1st, 2016 to December 31st, 2017; after the reform: January 1st, 2018 to December 31st, 2019). All interrupted time series analyses are fitted for a Prais-Winsten model with the Durbin-Waston statistic to adjust for autocorrelation. Outcomes were adjusted for the number of discharge cases, age, sex, Charlson Comorbidity Index of patients, and seasonality, with a robust standard error

**Fig. 2:**
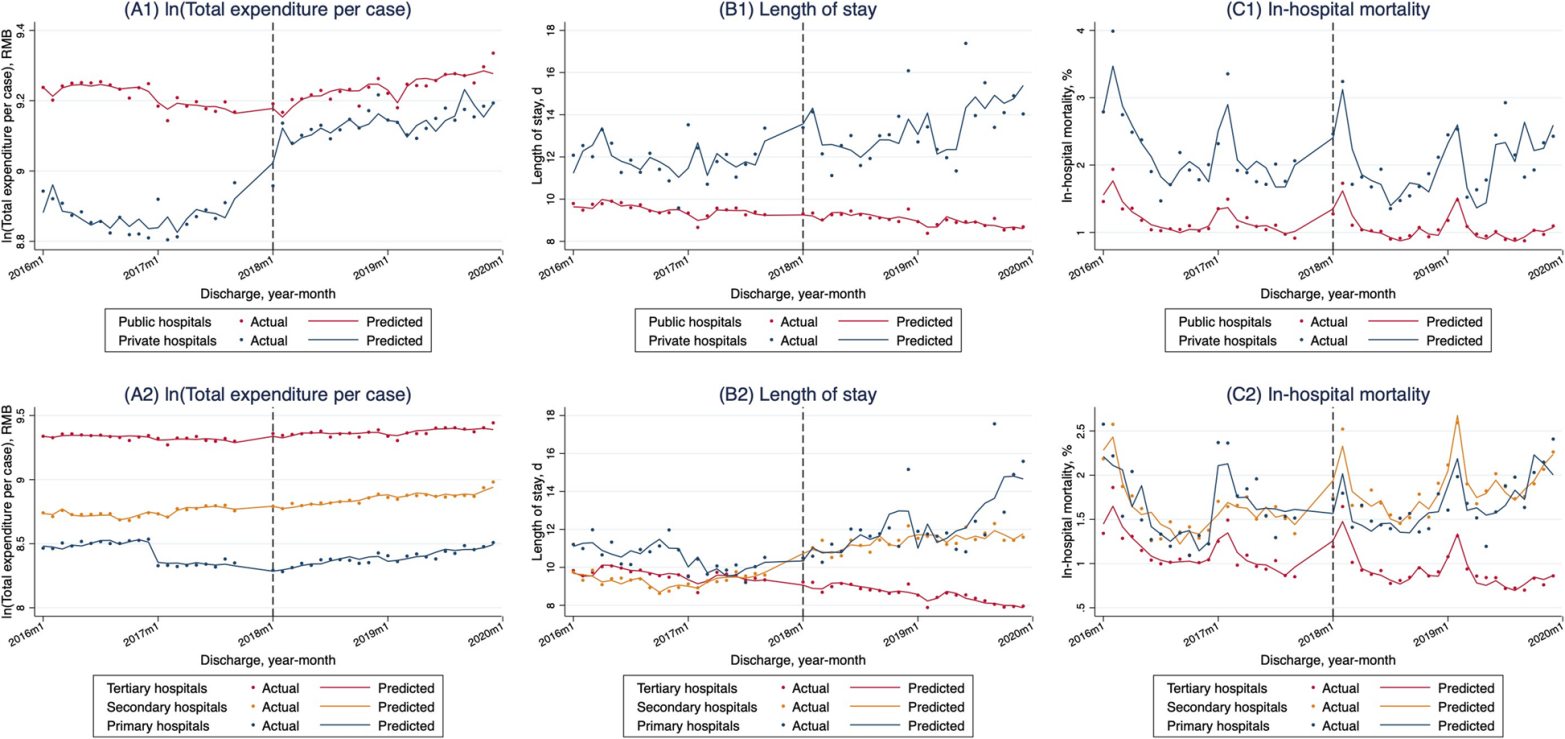
Monthly trends in adjusted total expenditure per case (in log form), length of stay, and in-hospital mortality rate of hospitalized insured patients in different hospitals. Notes: A1, Monthly trends of adjusted total expenditure per case (in log form and adjusted to 2019 considering inflation using the annual consumer price index of China) in different ownerships of hospitals; A2, in different levels of hospitals. B1, Monthly trends in the adjusted average length of stay in different ownerships of hospitals; B2, in different levels of hospitals. C1, Monthly trends in adjusted in-hospital mortality rate in different ownerships of hospitals; C2, in different levels of hospitals. The vertical dashed line denotes the implementation of the DIP payment reform on January 1st, 2018. The solid trend line is predicted based on segmented regression of the time series model (before the reform: January 1st, 2016 to December 31st, 2017; after the reform: January 1st, 2018 to December 31st, 2019). All interrupted time series analyses are fitted for a Prais-Winsten model with the Durbin-Waston statistic to adjust for autocorrelation. Outcomes were adjusted for the number of discharge cases, age, sex, Charlson Comorbidity Index of patients, and seasonality with a robust standard error

Table 2 presents the adjusted baseline level and trend as well as the change of level and monthly trend of outcome variables. In all hospitals (Table 2; Fig. 1A), the total expenditure per case declined significantly by 0.6% per month (*P* = 0.000) before the reform. After the reform, we found an immediate 5.9% increase (*P* = 0.001) and a 1.1% (*P* = 0.000) increase of the monthly trend in the total expenditure per case. As shown in Fig. 2A, the level of total expenditure per case was always higher in public hospitals and the highest in tertiary hospitals. We found similar significant immediate rises of total expenditure per case after the DIP reform in public hospitals (5.6%, *P* = 0.003), tertiary hospitals (7.4%, *P* = 0.000), and primary hospitals (6.4%, *P* = 0.006). We also found significant increases of monthly trends in public (1.1%, *P* = 0.000) hospitals and each level of hospitals (tertiary at 0.6%, *P* = 0.000; secondary at 0.4%, *P* = 0.047; primary at 0.9%, *P* = 0.039) hospitals. When also considering the hospital level in the analyses of different ownerships and visa versa, the change patterns after the reform kept the same with some results changing from significant to insignificant (Table S3).

**Table 2:** Interrupted time series (ITS) analyses for total expenditure per case, length of stay, and in-hospital mortality of hospitalized insured patients before and after the DIP reform

Indicators	Baseline monthly slope (β_1_)		Step change (β_2_)		Monthly slope change (β_3_)		Constant (β_0_)	
	Estimate (95%CI)	*P* value	Estimate (95%CI)	*P* value	Estimate (95%CI)	*P* value	Estimate (95%CI)	*P* value
**All hospitals**								
ln (Total expenditure per case)	-0.006 (-0.009, -0.003)	0.000	0.059 (0.025, 0.093)	0.001	0.011 (0.007, 0.014)	0.000	9.309 (8.296, 10.322)	0.000
Length of stay	-0.052 (-0.074, -0.031)	0.000	0.371 (0.128, 0.615)	0.004	0.009 (-0.017, 0.037)	0.492	-1.707 (-10.943, 7.528)	0.709
In-hospital mortality rate	-0.009 (-0.020, 0.002)	0.091	0.016 (-0.107, 0.140)	0.788	-0.003 (-0.015, 0.009)	0.622	-6.910 (-10.687, -3.133)	0.001
**Hospital ownership**								
Public hospitals								
ln (Total expenditure per case)	-0.006 (-0.009, -0.003)	0.000	0.056 (0.021, 0.092)	0.003	0.011 (0.008, 0.014)	0.000	9.690 (8.757, 10.623)	0.000
Length of stay	-0.060 (-0.079, -0.042)	0.000	0.309 (0.060, 0.558)	0.016	0.022 (0.001, 0.043)	0.041	-1.163 (-8.101, 5.775)	0.736
In-hospital mortality rate	-0.011 (-0.020, -0.002)	0.017	-0.017 (-0.129, 0.095)	0.766	0.001 (-0.009, 0.010)	0.901	-6.787 (-9.621, -3.953)	0.000
Private hospitals								
ln (Total expenditure per case)	0.003 (0.000, 0.007)	0.038	0.020 (-0.118, 0.157)	0.773	-0.003 (-0.010, 0.003)	0.322	8.601 (7.599, 9.602)	0.000
Length of stay	0.053 (-0.007, 0.113)	0.084	0.624 (-2.791, 4.039)	0.713	-0.141 (-0.290, 0.008)	0.062	-9.917 (-37.460, 17.627)	0.469
In-hospital mortality rate	0.005 (-0.019, 0.028)	0.697	-0.236 (-1.183, 0.712)	0.617	-0.083 (-0.133, -0.032)	0.002	1.589 (-4.179, 7.357)	0.579
**Hospital level**								
Tertiary hospitals								
ln (Total expenditure per case)	-0.002 (-0.005, -0.000)	0.033	0.074 (0.036, 0.113)	0.000	0.006 (0.004, 0.008)	0.000	9.597 (8.326, 10.869)	0.000
Length of stay	-0.050 (-0.068, -0.032)	0.000	0.051 (-0.254, 0.355)	0.737	-0.013 (-0.032, 0.005)	0.165	4.974 (-4.817, 14.764)	0.309
In-hospital mortality rate	-0.009 (-0.015, -0.003)	0.006	0.034 (-0.087, 0.154)	0.573	-0.003 (-0.010, 0.005)	0.452	-7.106 (-12.306, -1.906)	0.009
Secondary hospitals								
ln (Total expenditure per case)	-0.000 (-0.002, 0.001)	0.899	-0.049 (-0.092, -0.007)	0.025	0.004 (0.000, 0.008)	0.047	7.807 (7.328, 8.286)	0.000
Length of stay	-0.005 (-0.033, 0.023)	0.729	0.296 (-0.433, 1.025)	0.414	-0.048 (-0.105, 0.009)	0.095	12.784 (5.292, 20.276)	0.001
In-hospital mortality rate	0.005 (-0.006, 0.015)	0.349	-0.144 (-0.416, 0.129)	0.291	-0.037 (-0.059, -0.014)	0.002	-4.148 (-6.841, -1.455)	0.004
Primary hospitals								
ln (Total expenditure per case)	-0.005 (-0.011, 0.001)	0.113	0.064 (0.020, 0.109)	0.006	0.009 (0.000, 0.018)	0.039	9.373 (8.651, 10.095)	0.000
Length of stay	-0.100 (-0.262, 0.063)	0.222	1.096 (-0.167, 2.360)	0.087	0.235 (0.022, 0.449)	0.032	7.788 (-19.674, 35.251)	0.568
In-hospital mortality rate	-0.026 (-0.076, 0.023)	0.291	-0.261 (-0.609, 0.087)	0.137	0.066 (-0.010, 0.141)	0.088	-9.395 (-17.074, -1.716)	0.018

DIP denoted the Diagnosis-Intervention Packet payment reform; CI the confidence interval. Total expenditure was adjusted to 2019 considering inflation using the annual consumer price index of China. Total expenditure per case was logarithmically transformed in the ITS model. ITS analyses controlled for the number of discharge cases, age, sex, Charlson Comorbidity Index of patients, and seasonality, with robust standard errors

The increased trend of total expenditure per case was accompanied by a significantly decreased monthly trend for the number of discharge cases in the whole sample and almost every ownership and level of hospitals, which has been significantly increasing before the DIP reform (Figure S1A, Figure S2A, and Table S4). The change pattern of the monthly trend for RW per case was similar to total expenditure per case, which significantly increased after the DIP reform in the whole sample (10.147 per month, *P* = 0.000), public hospitals (9.379 per month, *P* = 0.000), tertiary hospitals (7.859 per month, *P* = 0.000) and secondary hospitals (5.673 per month, *P* = 0.000) (Figure S1B, Figure S2B, and Table S4).

### Average length of stay

The average LOS declined by 0.052 days per month significantly (*P* = 0.000) before the DIP reform in the whole sample (Table 2; Fig. 1B). The implementation of the DIP payment reform was associated with an immediate increase of 0.371 days (*P* = 0.004) and an insignificant change of the monthly trend. The level of average LOS only increased significantly following the DIP reform in public hospitals (0.309 days, *P* = 0.016). And the monthly trend significantly increased in public hospitals (0.022 days, *P* = 0.041) and primary hospitals (0.235 days, *P* = 0.032) after the DIP payment reform (Table 2; Fig. 2B). When considering hospital level in the analyses of different ownerships and visa versa, the change patterns of immediate level and monthly trend for LOS kept the same with the significance of some results changed (Table S3).

### In-hospital mortality rate

The overall in-hospital mortality rate showed an insignificant descending monthly trend before the DIP payment reform (Table 2; Fig. 1C). The level and trend remained unchanged after the reform. Before the DIP reform, the in-hospital mortality rate has been significantly decreasing in public hospitals (0.011 percentage points, *P* = 0.017) and tertiary hospitals (0.009 percentage points, *P* = 0.006). And it showed a significantly decreasing monthly trend in private and secondary hospitals (0.083 percentage points, *P* = 0.002; 0.037 percentage points, *P* = 0.002, respectively) after the reform (Table 2; Fig. 2C). The change patterns of in-hospital mortality were consistent when considering hospital level in the analyses of different ownerships and visa versa, while the significance of some results changed (Table S3).

## Discussion

To the best of our knowledge, this is the first empirical study to compare the impact of the DIP payment reform under the regional global budget on the total expenditure per case, LOS, and in-hospital mortality rate across hospitals with different ownership (public, private) and different accreditation level (tertiary, secondary, and primary). The ITS analysis showed that the DIP payment reform resulted in a significant increase in the monthly trend of adjusted total expenditure per case in public hospitals, tertiary hospitals, secondary hospitals, and primary hospitals. Additionally, after the DIP reform in our studied city, the monthly trend of LOS increased significantly in public and primary hospitals. Further, the monthly trend of in-hospital mortality rate decreased significantly in private and secondary hospitals. When considering the interaction between the two dimensions of hospital ownership and the level in robustness check, the change patterns of the monthly trend remained consistent with our previous findings.

Public and private hospitals responded inconsistently to the DIP payment reform. The monthly trend of total expenditure per case has increased in public hospitals after the DIP payment reform, while decreasing in private hospitals with no significance. Several factors may contribute to this phenomenon. Firstly, after the DIP reform, public hospitals were motivated to obtain higher RW by treating more serious patients that require high treatment intensity, while private hospitals lacking corresponding capacity may prioritize cost-saving measures by accepting patients with routine diagnoses and treatments. This was also confirmed by our findings that the monthly trend for the number of discharge cases decreased, and the monthly trend for RW per case increased in public hospitals after the DIP reform. Secondly, under the requirement of public welfare, and for the development and construction of key disciplines, public hospitals have the responsibility and ability to treat high-severity patients who require high treatment intensity, while private hospitals have financial incentives to treat low-severity patients. Evidence from England and France on cherry picking illustrates that private providers have been found to treat less complex patients than public hospitals [22, 23]. Thirdly, compared with public hospitals, private hospitals may have stronger financial incentives to shift more inpatient services to outpatient services where patients are separately reimbursed to reduce the cost of inpatient services after the participation of the DIP reform [24]. Previous research also supports this point, as it reported that patients admitted to private hospitals had significantly increased odds of being transferred to the hospital’s outpatient unit relative to public hospitals (ORs = 5.11, 14.69, and 27.62, respectively, for cesarean section, hernia, and hemorrhoidectomy operations) [8, 25].

Our results showed that the previously descending monthly trend of LOS changed slower significantly in public hospitals while decreasing in private hospitals after the DIP reform. Additionally, the monthly trend of in-hospital mortality rate in private hospitals decreased significantly, whereas the previously descending monthly trend of in-hospital mortality rate remain unchanged in public hospitals after the DIP reform. The main reason for these findings may be that in our study city, the LOS and in-hospital mortality rates of private hospitals were initially higher than those of public hospitals. However, these indicators had significantly decreased in public hospitals prior to the DIP reform, leaving less potential for further improvement in public hospitals after the reform. Furthermore, our findings support previous research that demonstrated hospital ownership was a significant determinant of hospital profitability under a case-based payment system [26]. Unlike public hospitals, which were financially supported by government funding or philanthropic donations, private hospitals were more willing to seek any feasible means to increase revenues to stay competitive in the healthcare market. Private hospitals have stronger motivation to compete for the limited medical reimbursement fund and benefit from the profits, especially as our study city moved from the “fixed-rate per admission with a cap on annual compensation” policy to the DIP payment under the regional global budget. Consequently, private hospitals have stronger financial incentives to improve efficiency by shortening the LOS and improving their reputation and the quality of care by reducing in-hospital mortality.

The results that the total expenditure per case in high-level hospitals increased higher than that of lower-level hospitals were consistent with our expectations. After the DIP reform, the actual payments per inpatient case shifted from the “fixed-rate per admission with a cap on annual compensation” to the floating reimbursements depending on the pre-determined annual regional global budget and the sum of RW points for all inpatient cases, thus the demand for medical services may be released. Under the context of the DIP reform, hospitals can obtain higher DIP points and reimbursements by treating patients with severe illnesses and providing highly intensive treatment, while tertiary hospitals combining the use of advanced technology and medical resources are more capable of selecting and treating such patients than lower-level hospitals [27]. Higher illness severity with highly intensive treatment undoubtedly results in higher expenditure per case in high-level hospitals [28]. Our findings also confirmed that CCI in high-level hospitals was higher than that in lower-level hospitals, and it increased significantly in tertiary hospitals and secondary hospitals after the reform. Our results may imply that DIP reform encourages high-level hospitals to treat patients with higher illness severities. Future researchers should pay more attention to whether the increase in the cost expenditure in high-level hospitals is reasonable under DIP payment long-term reform. Additionally, we found that the monthly trend of LOS decreased in tertiary hospitals after the DIP reform, and the change pattern of the monthly trend of LOS was significant when controlling for hospital ownership. The reason may be that tertiary hospitals with refined management and advanced information systems were more likely to improve efficiency than primary hospitals.

### Limitations

This study has some limitations. First, our study only investigated the short-term effects of the DIP payment reform across different hospitals. Future research is needed to evaluate the responses across different types of hospitals to the DIP policies in the long term. Second, this study did not consider the potential mechanisms of different outcomes across different hospitals after the reform, such as changes in disease structure among different hospitals, which needs further exploration in the future. Third, researchers should be cautious about extending these empirical results to other regions, because our selected city implemented a “fixed rate per admission with a cap on annual compensation” policy before DIP payment reform. Fourth, the ITS of this study does not set up a parallel control group during the same period due to the lack of a suitable control group, so it may be difficult to make causal statements about the reform. Additionally, we exclude data from October to December 2017 before the reform due to the relatively more missing data and small sample size in the original dataset.

## Conclusions

Our study demonstrated that implementing the DIP payment reform under the regional global budget would yield inconsistent consequences across different hospitals. DIP payment reform encouraged public hospitals and high-level hospitals to treat patients with higher illness severities and requiring high treatment intensity, resulting in a significant increase in total expenditure per case. The inconsistencies between public and private hospitals may be attributed to their different baseline levels prior to the reform and their different responses to the incentives created by the reform. Our findings remind policymakers that they need to consider inconsistent consequences across different hospitals when seeking to expand the new DIP payment system to other regions.

## Supplementary Information

**Additional file 1:** **Table S1.** Sample characteristics of hospitalized insured patients by hospital ownership, 2016-2019. **Table S2.** Sample characteristics of hospitalized insured patients by hospital level, 2016-2019. **Table S3.** Interrupted time series (ITS) analyses for total expenditure per case, length of stay, and in-hospital mortality of hospitalized insured patients before and after the DIP reform (considering the relationship between hospital ownership and level). **Figure S1.** Monthly trends in adjusted discharge cases and relative weight per case of hospitalized insured patients. Notes: A, Monthly trends in adjusted discharge cases. B, Monthly trends in adjusted relative weight per case. The vertical dashed line denotes the implementation of the DIP payment reform on January 1^st^, 2018. The solid trend line is predicted based on segmented regression of the time series model (before the reform: January 1^st^, 2016 to December 31^st^, 2017; after the reform: January 1^st^, 2018 to December 31^st^, 2019). All interrupted time series analyses are fitted for a Prais-Winsten model with the Durbin-Waston statistic to adjust for autocorrelation. Discharge cases were adjusted for seasonality, and relative weight per case was adjusted for the number of discharge cases, age, sex, Charlson Comorbidity Index of patients, and seasonality, both with a robust standard error. **Figure S2.** Monthly trends in adjusted discharge cases and relative weight per case of hospitalized insured patients in different hospitals. Notes: A1, Monthly trends of adjusted discharge cases in different ownerships of hospitals; A2, in different levels of hospitals. B1, Monthly trends in adjusted relative weight per case in different ownerships of hospitals; B2, in different levels of hospitals. The vertical dashed line denotes the implementation of the DIP payment reform on January 1^st^, 2018. The solid trend line is predicted based on segmented regression of the time series model (before the reform: January 1^st^, 2016 to December 31^st^, 2017; after the reform: January 1^st^, 2018 to December 31^st^, 2019). All interrupted time series analyses are fitted for a Prais-Winsten model with the Durbin-Waston statistic to adjust for autocorrelation. Discharge cases were adjusted for seasonality, and relative weight per case was adjusted for the number of discharge cases, age, sex, Charlson Comorbidity Index of patients, and seasonality, both with a robust standard error. **Table S4.** Interrupted time series (ITS) analyses for discharge cases and relative weight per case of hospitalized insured patients before and after the DIP reform.

## Data Availability

The data analyzed during the current study are available from Guangzhou Healthcare Security Administration but restrictions apply to the availability of these data, which were not publicly available due to we used under the license for the current study. All questions regarding the availability of the data should be directed to the corresponding author.

## Abbreviations

DIP: Diagnosis-intervention packet
LOS: Length of stay
FFS: Fee-for-service
PPS: Prospective payment system
DRGs: Diagnosis Related Groups
CCI: Charlson Comorbidity Index
ITS: Interrupted time series
RW: Relative weight
