# Supplementary material for: Variations in the impact of the new case-based payment reform on medical costs, length of stay, and quality across different hospitals in China: an interrupted time series analysis
Source: BMC Health Serv Res. 2023 Jun 2;23:568. doi: 10.1186/s12913-023-09553-x (PMC10236872; doi:10.1186/s12913-023-09553-x)
Supplement: Supplementary file 1 — Additional file 1: Table S1. Sample characteristics of hospitalized insured patients by hospital ownership, 2016-2019. Table S2. Sample characteristics of hospitalized insured patients by hospital level, 2016-2019. Table S3. Interrupted time series (ITS) analyses for total expenditure per case, length of stay, and in-hospital mortality of hospitalized insured patients before and after the DIP reform (considering the relationship between hospital ownership and level). Figure S1. Monthly trends in adjusted discharge cases and relative weight per case of hospitalized insured patients. Notes: A, Monthly trends in adjusted discharge cases. B, Monthly trends in adjusted relative weight per case. The vertical dashed line denotes the implementation of the DIP payment reform on January 1st, 2018. The solid trend line is predicted based on segmented regression of the time series model (before the reform: January 1st, 2016 to December 31st, 2017; after the reform: January 1st, 2018 to December 31st, 2019). All interrupted time series analyses are fitted for a Prais-Winsten model with the Durbin-Waston statistic to adjust for autocorrelation. Discharge cases were adjusted for seasonality, and relative weight per case was adjusted for the number of discharge cases, age, sex, Charlson Comorbidity Index of patients, and seasonality, both with a robust standard error. Figure S2. Monthly trends in adjusted discharge cases and relative weight per case of hospitalized insured patients in different hospitals. Notes: A1, Monthly trends of adjusted discharge cases in different ownerships of hospitals; A2, in different levels of hospitals. B1, Monthly trends in adjusted relative weight per case in different ownerships of hospitals; B2, in different levels of hospitals. The vertical dashed line denotes the implementation of the DIP payment reform on January 1st, 2018. The solid trend line is predicted based on segmented regression of the time series model (before the reform: January 1st, 2 [file 12913_2023_9553_MOESM1_ESM.docx]

**Supplementary Appendix**

Table S1 Sample characteristics of hospitalized insured patients by hospital ownership, 2016-2019.

| Variables | Before DIP reform, 2016-2017 | After DIP reform, 2018-2019 | *P* value |
| --- | --- | --- | --- |
| **Public hospital (N=235)** |  |  |  |
| Patient characteristics |  |  |  |
| Discharge cases, No. | 1,988,492 | 2,832,538 |  |
| Age, mean (SD) | 58.18 (18.18) | 58.47 (18.16) | 0.000 |
| Male sex, No. (%) | 900,001 (45.26) | 1,286,922 (45.43) | 0.000 |
| Charlson Comorbidity Index, mean (SD) | 0.76 (1.35) | 0.94 (1.50) | 0.000 |
| Hospital level, No. (%) |  |  | 0.000 |
| Tertiary (N=82) | 1,609,386 (80.93) | 2,271,739 (80.20) |  |
| Secondary (N=70) | 300,959 (15.14) | 395,443 (13.96) |  |
| Primary (N=95) | 78,147 (3.93) | 165,356 (5.84) |  |
| Patient outcomes |  |  |  |
| Total expenditure per case, mean (SD), RMB | 15668.39 (19677.84) | 16059.39 (19602.01) | 0.000 |
| Length of stay, mean (SD), d | 9.49 (10.98) | 9.01 (10.85) | 0.000 |
| In-hospital mortality, mean (SD), % | 1.16 (10.73) | 1.05 (10.18) | 0.000 |
| **Private hospital (N=59)** |  |  |  |
| Patient characteristics |  |  |  |
| Discharge cases, No. | 88,663 | 151,433 |  |
| Age, mean (SD) | 63.39 (18.80) | 62.46 (18.52) | 0.000 |
| Male sex, No. (%) | 34,406 (38.81) | 71,394 (47.15) | 0.000 |
| Charlson Comorbidity Index, mean (SD) | 0.72 (1.20) | 0.80 (1.36) | 0.000 |
| Hospital level, No. (%) |  |  | 0.000 |
| Tertiary (N=8) | 61,337 (69.18) | 93,396 (61.67) |  |
| Secondary (N=22) | 16,349 (18.44) | 36,180 (23.89) |  |
| Primary (N=31) | 10,977 (12.38) | 21,857 (14.43) |  |
| Patient outcomes |  |  |  |
| Total expenditure per case, mean (SD), RMB | 10469.53 (15335.66) | 13135.13 (14950.33) | 0.000 |
| Length of stay, mean (SD), d | 11.79 (17.78) | 13.06 (22.41) | 0.000 |
| In-hospital mortality, mean (SD), % | 2.08 (14.26) | 1.94 (13.79) | 0.021 |
| Notes: DIP denoted the Diagnosis-Intervention Packet payment reform; N the number of hospitals. Total expenditure was adjusted to 2019 considering inflation using the annual consumer price index of China. The total number of tertiary, secondary, and primary hospitals in public hospitals exceeds the number of public hospitals because of the level changes of some hospitals during the study period. Specifically, 12 public hospitals experienced level changes, including four hospitals changing from primary to secondary, six from secondary to tertiary, one from secondary to primary, and one from tertiary to secondary. The number of cases in these hospitals was 114,237, accounting for 2.37% of the whole sample of public hospitals. Similarly, two private hospitals experienced level changes, which both changed from primary to secondary during the study period. The number of cases in the two hospitals was 4,180, accounting for 1.74% of the whole sample of private hospitals. | | | |

Table S2 Sample characteristics of hospitalized insured patients by hospital level, 2016-2019.

| Variables | Before DIP reform, 2016-2017 | After DIP reform, 2018-2019 | *P* value |
| --- | --- | --- | --- |
| **Tertiary hospital (N=90)** |  |  |  |
| Patient characteristics |  |  |  |
| Discharge cases, No. | 1,670,723 | 2,365,135 |  |
| Age, mean (SD) | 57.40 (17.46) | 56.94 (17.43) | 0.000 |
| Male sex, No. (%) | 777,912 (46.56) | 1,094,838 (46.29) | 0.000 |
| Charlson Comorbidity Index, mean (SD) | 0.81 (1.41) | 0.99 (1.57) | 0.000 |
| Hospital ownership, No. (%) |  |  | 0.000 |
| Public (N=82) | 1,609,386 (96.33) | 2,271,739 (96.05) |  |
| Private (N=8) | 61,337 (3.67) | 93,396 (3.95) |  |
| Patient outcomes |  |  |  |
| Total expenditure per case, mean (SD), RMB | 17330.77 (20883.98) | 17941.14 (20825.71) | 0.000 |
| Length of stay, mean (SD), d | 9.60 (10.81) | 8.60 (9.48) | 0.000 |
| In-hospital mortality, mean (SD), % | 1.10 (10.44) | 0.91 (9.51) | 0.000 |
| **Secondary hospital (N=92)** |  |  |  |
| Patient characteristics |  |  |  |
| Discharge cases, No. | 317,308 | 431,623 |  |
| Age, mean (SD) | 61.04 (20.61) | 63.93 (19.87) | 0.000 |
| Male sex, No. (%) | 121,377 (38.25) | 181,355 (42.02) | 0.000 |
| Charlson Comorbidity Index, mean (SD) | 0.57 (0.98) | 0.77 (1.19) | 0.000 |
| Hospital ownership, No. (%) |  |  | 0.000 |
| Public (N=70) | 300,959 (94.85) | 395,443 (91.62) |  |
| Private (N=22) | 16,349 (5.15) | 36,180 (8.38) |  |
| Patient outcomes |  |  |  |
| Total expenditure per case, mean (SD), RMB | 8373.39 (10005.58) | 9299.67 (10158.63) | 0.000 |
| Length of stay, mean (SD), d | 9.31 (12.44) | 11.34 (16.69) | 0.000 |
| In-hospital mortality, mean (SD), % | 1.59 (12.51) | 1.83 (13.42) | 0.000 |
| **Primary hospital (N=126)** |  |  |  |
| Patient characteristics |  |  |  |
| Discharge cases, No. | 89,124 | 187,213 |  |
| Age, mean (SD) | 67.91 (19.67) | 68.33 (18.30) | 0.000 |
| Male sex, No. (%) | 35,118 (39.40) | 82,123 (43.87) | 0.000 |
| Charlson Comorbidity Index, mean (SD) | 0.63 (0.95) | 0.58 (0.92) | 0.000 |
| Hospital ownership, No. (%) |  |  | 0.000 |
| Public (N=95) | 78,147 (87.68) | 165,356 (88.33) |  |
| Private (N=31) | 10,977 (12.32) | 21,857 (11.67) |  |
| Patient outcomes |  |  |  |
| Total expenditure per case, mean (SD), RMB | 5478.55 (5334.94) | 5840.12 (6402.61) | 0.000 |
| Length of stay, mean (SD), d | 10.30 (16.43) | 12.09 (19.96) | 0.000 |
| In-hospital mortality, mean (SD), % | 1.69 (12.88) | 1.64 (12.71) | 0.390 |
| Notes: DIP denoted the Diagnosis-Intervention Packet payment reform; N the number of hospitals. Total expenditure was adjusted to 2019 considering inflation using the annual consumer price index of China. | | | |

Table S3 Interrupted time series (ITS) analyses for total expenditure per case, length of stay, and in-hospital mortality of hospitalized insured patients before and after the DIP reform (considering the relationship between hospital ownership and level).

| Indicators | Baseline monthly slope (β_1_) | |  | Step change (β_2_) | |  | Monthly slope change (β_3_) | |  | Constant (β_0_) | |
| --- | --- | --- | --- | --- | --- | --- | --- | --- | --- | --- | --- |
|  | Estimate (95%CI) | *P* value |  | Estimate (95%CI) | *P* value |  | Estimate (95%CI) | *P* value |  | Estimate (95%CI) | *P* value |
| **Hospital ownership** |  |  |  |  |  |  |  |  |  |  |  |
| Public hospitals |  |  |  |  |  |  |  |  |  |  |  |
| ln (Total expenditure per case) | -0.000 (-0.005, 0.005) | 0.929 |  | 0.033 (-0.007, 0.074) | 0.106 |  | 0.005 (-0.000, 0.010) | 0.061 |  | 8.889 (7.852, 9.927) | 0.000 |
| Length of stay | -0.057 (-0.090, -0.025) | 0.001 |  | 0.178 (-0.062, 0.417) | 0.141 |  | 0.032 (-0.007, 0.071) | 0.109 |  | 0.300 (-6.805, 7.406) | 0.932 |
| In-hospital mortality rate | -0.026 (-0.042, -0.010) | 0.003 |  | 0.024 (-0.085, 0.132) | 0.660 |  | 0.017 (0.001, 0.033) | 0.044 |  | -4.989 (-7.837, -2.141) | 0.001 |
| Private hospitals |  |  |  |  |  |  |  |  |  |  |  |
| ln (Total expenditure per case) | 0.003 (-0.001, 0.006) | 0.111 |  | -0.003 (-0.144, 0.138) | 0.962 |  | -0.001 (-0.009, 0.007) | 0.809 |  | 8.636 (7.658, 9.613) | 0.000 |
| Length of stay | 0.017 (-0.059, 0.094) | 0.645 |  | -1.567 (-5.120, 1.987) | 0.376 |  | -0.108 (-0.283, 0.067) | 0.218 |  | -15.237 (-42.284, 11.810) | 0.260 |
| In-hospital mortality rate | 0.003 (-0.023, 0.029) | 0.803 |  | -0.289 (-1.381, 0.803) | 0.594 |  | -0.066 (-0.136, 0.005) | 0.067 |  | 2.174 (-3.586, 7.934) | 0.448 |
| **Hospital level** |  |  |  |  |  |  |  |  |  |  |  |
| Tertiary hospitals |  |  |  |  |  |  |  |  |  |  |  |
| ln (Total expenditure per case) | -0.003 (-0.005, - 0.001) | 0.008 |  | 0.087 (0.056, 0.118) | 0.000 |  | 0.003 (0.001, 0.005) | 0.012 |  | 8.551 (7.314, 9.788) | 0.000 |
| Length of stay | -0.054 (-0.069, -0.039) | 0.000 |  | 0.148 (-0.102, 0.399) | 0.237 |  | -0.036 (-0.052, -0.020) | 0.000 |  | -3.368 (-14.458, 7.721) | 0.541 |
| In-hospital mortality rate | -0.007 (-0.014, -0.000) | 0.041 |  | 0.016 (-0.108, 0.140) | 0.793 |  | -0.000 (-0.011, 0.010) | 0.940 |  | -6.798 (-11.911, -1.684) | 0.011 |
| Secondary hospitals |  |  |  |  |  |  |  |  |  |  |  |
| ln (Total expenditure per case) | -0.000 (-0.002, 0.001) | 0.663 |  | -0.064 (-0.116, -0.011) | 0.020 |  | 0.004 (-0.000, 0.008) | 0.071 |  | 8.247 (7.498, 8.995) | 0.000 |
| Length of stay | -0.005 (-0.033, 0.023) | 0.732 |  | 0.298 (-0.611, 1.207) | 0.510 |  | -0.048 (-0.107, 0.011) | 0.110 |  | 12.735 (-6.771, 32.241) | 0.193 |
| In-hospital mortality rate | 0.005 (-0.006, 0.015) | 0.357 |  | -0.142 (-0.426, 0.141) | 0.314 |  | -0.037 (-0.060, -0.013) | 0.003 |  | -4.199 (-8.883, 0.485) | 0.077 |
| Primary hospitals |  |  |  |  |  |  |  |  |  |  |  |
| ln (Total expenditure per case) | -0.002 (-0.007, 0.003) | 0.494 |  | 0.019 (-0.033, 0.071) | 0.459 |  | 0.006 (-0.002, 0.013) | 0.125 |  | 9.180 (8.373, 9.987) | 0.000 |
| Length of stay | -0.085 (-0.247, 0.076) | 0.290 |  | 0.822 (-0.390, 2.033) | 0.177 |  | 0.222 (0.006, 0.437) | 0.044 |  | 7.019 (-22.127, 36.164) | 0.627 |
| In-hospital mortality rate | -0.051 (-0.100, -0.002) | 0.042 |  | 0.142 (-0.304, 0.588) | 0.521 |  | 0.094 (0.027, 0.162) | 0.008 |  | -8.524 (-16.258, -0.790) | 0.032 |
| Notes: DIP denoted the Diagnosis-Intervention Packet payment reform; CI the confidence interval. Total expenditure was adjusted to 2019 considering inflation using the annual consumer price index of China. Total expenditure per case was logarithmically transformed in the ITS model. ITS analyses for hospital ownership controlled for the number of discharge cases, age, sex, Charlson Comorbidity Index of patients, hospital level, and seasonality, with robust standard errors. ITS analyses for hospital level controlled for the number of discharge cases, age, sex, Charlson Comorbidity Index of patients, hospital ownership, and seasonality, with robust standard errors. | | | | | | | | | | | |


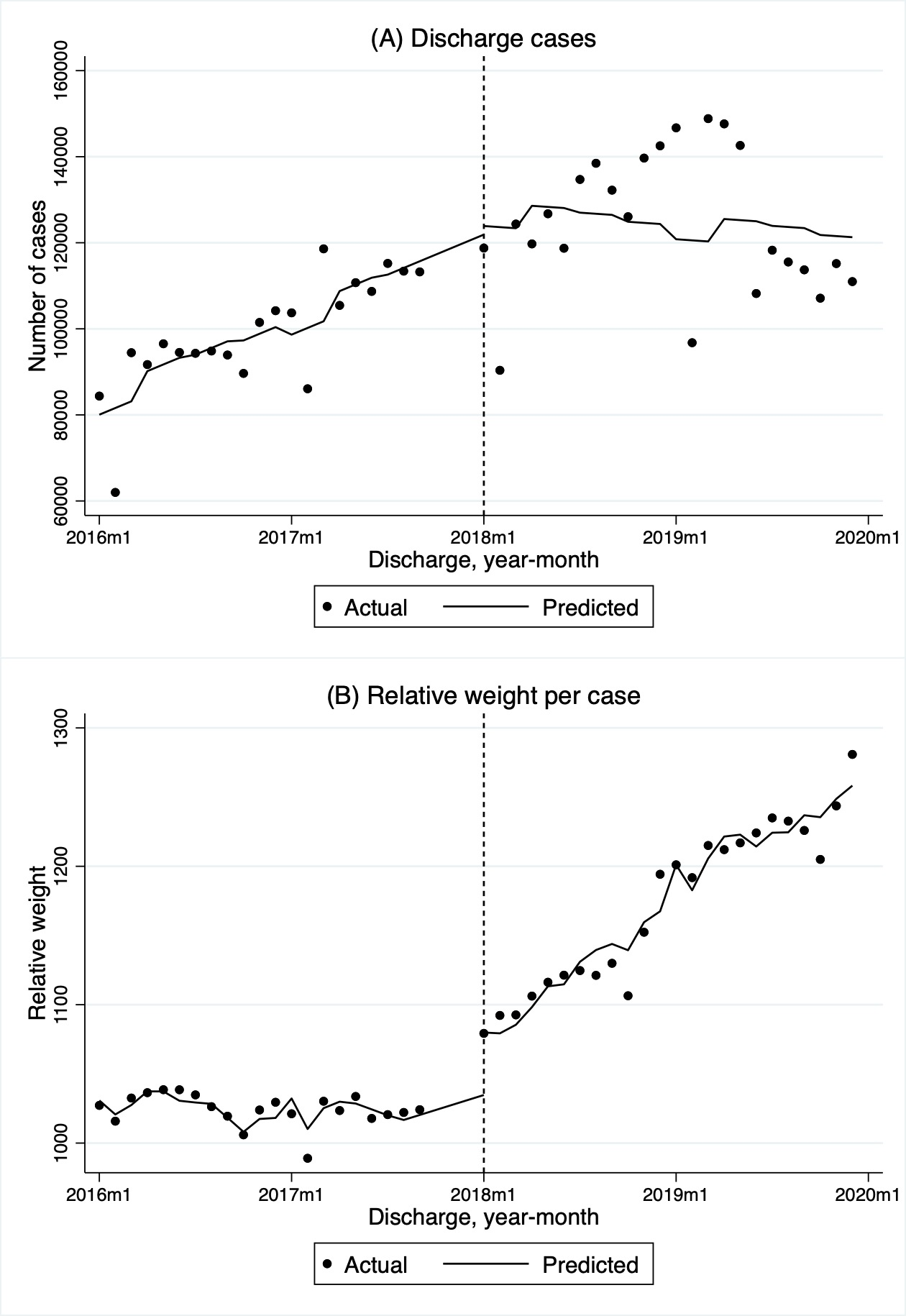


Figure S1 Monthly trends in adjusted discharge cases and relative weight per case of hospitalized insured patients.

Notes: A, Monthly trends in adjusted discharge cases. B, Monthly trends in adjusted relative weight per case. The vertical dashed line denotes the implementation of the DIP payment reform on January 1^st^, 2018. The solid trend line is predicted based on segmented regression of the time series model (before the reform: January 1^st^, 2016 to December 31^st^, 2017; after the reform: January 1^st^, 2018 to December 31^st^, 2019). All interrupted time series analyses are fitted for a Prais-Winsten model with the Durbin-Waston statistic to adjust for autocorrelation. Discharge cases were adjusted for seasonality, and relative weight per case was adjusted for the number of discharge cases, age, sex, Charlson Comorbidity Index of patients, and seasonality, both with a robust standard error.


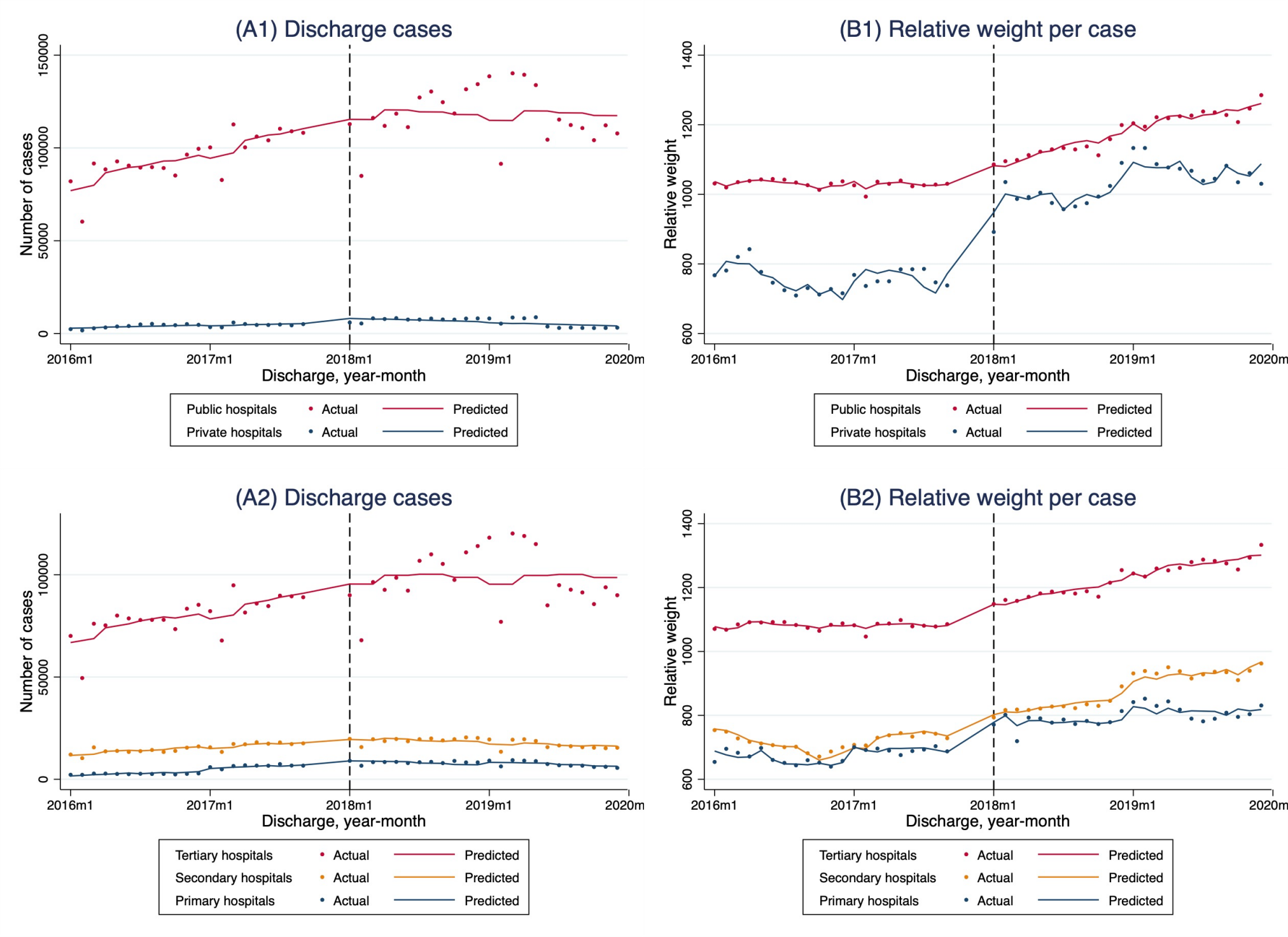


Figure S2. Monthly trends in adjusted discharge cases and relative weight per case of hospitalized insured patients in different hospitals.

Notes: A1, Monthly trends of adjusted discharge cases in different ownerships of hospitals; A2, in different levels of hospitals. B1, Monthly trends in adjusted relative weight per case in different ownerships of hospitals; B2, in different levels of hospitals. The vertical dashed line denotes the implementation of the DIP payment reform on January 1^st^, 2018. The solid trend line is predicted based on segmented regression of the time series model (before the reform: January 1^st^, 2016 to December 31^st^, 2017; after the reform: January 1^st^, 2018 to December 31^st^, 2019). All interrupted time series analyses are fitted for a Prais-Winsten model with the Durbin-Waston statistic to adjust for autocorrelation. Discharge cases were adjusted for seasonality, and relative weight per case was adjusted for the number of discharge cases, age, sex, Charlson Comorbidity Index of patients, and seasonality, both with a robust standard error.

Table S4 Interrupted time series (ITS) analyses for discharge cases and relative weight per case of hospitalized insured patients before and after the DIP reform.

| Indicators | Baseline monthly slope (β_1_) | |  | Step change (β_2_) | |  | Monthly slope change (β_3_) | |  | Constant (β_0_) | |
| --- | --- | --- | --- | --- | --- | --- | --- | --- | --- | --- | --- |
|  | Estimate (95%CI) | *P* value |  | Estimate (95%CI) | *P* value |  | Estimate (95%CI) | *P* value |  | Estimate (95%CI) | *P* value |
| **All hospitals** |  |  |  |  |  |  |  |  |  |  |  |
| Number of discharge cases | 1550.141 (797.785, 2302.498) | 0.000 |  | 6632.723 (-9717.541, 22982.990) | 0.417 |  | -1806.228 (-3001.784, -610.671) | 0.004 |  | 80056.190 (67700.530, 92411.850) | 0.000 |
| Relative weight per case | -2.563 (-4.278, -0.848) | 0.005 |  | 51.782 (30.244, 73.321) | 0.000 |  | 10.147 (8.025, 12.269) | 0.000 |  | 695.149 (94.191, 1296.107) | 0.025 |
| **Hospital ownership** |  |  |  |  |  |  |  |  |  |  |  |
| Public hospitals |  |  |  |  |  |  |  |  |  |  |  |
| Number of discharge cases | 1454.403 (789.438, 2119.367) | 0.000 |  | 3491.884 (-10839.780, 17823.550) | 0.625 |  | -1497.927 (-2550.731, -445.122) | 0.006 |  | 76983.680 (65804.770, 88162.590) | 0.000 |
| Relative weight per case | -2.015 (-3.802, -0.228) | 0.028 |  | 49.188 (25.055, 73.322) | 0.000 |  | 9.379 (7.251, 11.507) | 0.000 |  | 947.921 (352.323, 1543.519) | 0.003 |
| Private hospitals |  |  |  |  |  |  |  |  |  |  |  |
| Number of discharge cases | 106.518 (3.700, 209.336) | 0.043 |  | 2696.512 (-206.519, 5599.543) | 0.068 |  | -300.974 (-504.778, -97.170) | 0.005 |  | 2885.213 (1846.443, 3923.984) | 0.000 |
| Relative weight per case | 1.468 (-1.878, 4.814) | 0.379 |  | 64.381 (-59.013, 187.776) | 0.296 |  | 3.517 (-2.829, 9.862) | 0.268 |  | 96.942 (-687.095, 880.980) | 0.803 |
| **Hospital level** |  |  |  |  |  |  |  |  |  |  |  |
| Tertiary hospitals |  |  |  |  |  |  |  |  |  |  |  |
| Number of discharge cases | 961.917 (291.015, 1632.818) | 0.006 |  | 5510.722 (-8649.521, 19670.970) | 0.436 |  | -968.185 (-2011.214, 74.843) | 0.068 |  | 66856.680 (55962.490, 77750.870) | 0.000 |
| Relative weight per case | -0.780 (-2.163, 0.603) | 0.260 |  | 60.363 (40.376, 80.351) | 0.000 |  | 7.859 (6.302, 9.416) | 0.000 |  | 701.026 (17.578, 1384.475) | 0.045 |
| Secondary hospitals |  |  |  |  |  |  |  |  |  |  |  |
| Number of discharge cases | 279.184 (194.674, 363.694) | 0.000 |  | 1105.146 (-476.162, 2686.453) | 0.165 |  | -470.050 (-593.058, -347.041) | 0.000 |  | 11678.670 (10141.430, 13215.920) | 0.000 |
| Relative weight per case | 0.293 (-0.953, 1.540) | 0.635 |  | 19.706 (-12.548, 51.960) | 0.223 |  | 5.673 (2.993, 8.353) | 0.000 |  | -102.162 (-335.008, 130.684) | 0.379 |
| Primary hospitals |  |  |  |  |  |  |  |  |  |  |  |
| Number of discharge cases | 304.297 (249.506, 359.088) | 0.000 |  | 70.099 (-1280.959, 1421.157) | 0.917 |  | -363.718 (-451.474, -275.961) | 0.000 |  | 1613.883 (942.312, 2285.455) | 0.000 |
| Relative weight per case | 1.417 (-2.183, 5.017) | 0.429 |  | 73.705 (48.473, 98.937) | 0.000 |  | 0.804 (-4.125, 5.733) | 0.742 |  | -178.464 (-819.878, 462.949) | 0.575 |
| Notes: DIP denoted the Diagnosis-Intervention Packet payment reform; CI the confidence interval. ITS analyses controlled for seasonality in the analysis of the number of discharge cases and controlled for the number of discharge cases, age, sex, Charlson Comorbidity Index of patients, and seasonality in the analysis of relative weight per case, both with robust standard errors. | | | | | | | | | | | |
